# Numerical analysis of thermal conductive hybrid nanofluid flow over the surface of a wavy spinning disk

**DOI:** 10.1038/s41598-020-75905-w

**Published:** 2020-11-02

**Authors:** Ali Ahmadian, Muhammad Bilal, Muhammad Altaf Khan, Muhammad Imran Asjad

**Affiliations:** 1 Institute of IR 4.0, The National University of Malaysia (UKM), 43600 Bangi, Selangor Malaysia; 2 Department of Mathematics, City University of Science and Information Technology, Peshawar, Pakistan; 3 Informetrics Research Group, Ton Duc Thang University, Ho Chi Minh City, Vietnam; 4 Faculty of Mathematics and Statistics, Ton Duc Thang University, Ho Chi Minh City, Vietnam; 5 Department of Mathematics, University of Management and Technology Lahore, Lahore, Pakistan

**Keywords:** Mathematics and computing, Applied mathematics

## Abstract

A three dimensional (3D) numerical solution of unsteady, Ag-MgO hybrid nanoliquid flow with heat and mass transmission caused by upward/downward moving of wavy spinning disk has been scrutinized. The magnetic field has been also considered. The hybrid nanoliquid has been synthesized in the presence of Ag-MgO nanoparticles. The purpose of the study is to improve the rate of thermal energy transmission for several industrial purposes. The wavy rotating surface increases the heat transmission rate up to 15%, comparatively to the flat surface. The subsequent arrangement of modeled equations is diminished into dimensionless differential equation. The obtained system of equations is further analytically expounded via Homotopy analysis method HAM and the numerical Parametric continuation method (PCM) method has been used for the comparison of the outcomes. The results are graphically presented and discussed. It has been presumed that the geometry of spinning disk positively affects the velocity and thermal energy transmission. The addition of hybrid nanoparticles (silver and magnesium-oxide) significantly improved thermal property of carrier fluid. It uses is more efficacious to overcome low energy transmission. Such as, it provides improvement in thermal performance of carrier fluid, which play important role in power generation, hyperthermia, micro fabrication, air conditioning and metallurgical field.

## Introduction

The transmission of heat with the fluid flow has been a great area of research due to its wide application in the field of electronic devices and heat exchanger^1^. To escalate the heat transport and improve the flow pattern the extended surface is highly effective configuration. The heat transfer characteristic and fluid flow of the Sinusoidal-Corrugated channels are numerically investigated by Khoshvaght-Aliabadi^2^. The parameter effect including length of wave and amplitude, channel height and length, volume fraction of nanoparticle and Reynolds number were analyzed. Rashidi et al.^3^ tackled flow field and the heat transmission through a wavy channel. The uniform suction upshots deploy on the flow due to spinning disk is investigated by Stuart^4^. In early studies the heat transfer on rotating disk was examined by^5^. As flow started from rest, the steady motion of flow was obtained numerically by^6^. Kuiken^7^ clarified the blowing effect induced by porous rotating disk. Turkyilmazoglu^8^ reported the effect of stretching disk surface. Tabassum and Mustafa^9^ considered non-Newtonian Reiner-Rivlin fluid about rotating disk. Asifa et al.^10^ explores the behavior of an incompressible hybrid nanoliquid flow over an impermeable infinite spinning disk. Shuaib et al.^11^ has highlighted the 3D an incompressible fluid flow with heat transport over stretchable revolving disk.

Conventional fluid Such as, ethylene glycol, oil and water are play a conspicuous role in heat transfer, for example, in chemical processes, in cooling or heating processes, in power generation and in some other small electronics mechanism. But comparatively, the thermal energy transmission rate of these liquids very low and cannot accomplished the need of high rates of heat exchange. To overcome this deficiency, the nanometer-sized particles (1–100 nm), termed as nanoparticle is added to common fluid to enhance its thermal conductivity. The word ’nanofluid’ was first used by Choi^12^, which show high thermal conductivity, better rheological properties and stability as compared to fluid having micronized particles. The researcher used a variety of technique to prepare different types of nanoparticle to calculate the thermo physical properties^13^. Due to possessing the ability of dispersing and oil wetting nanoparticle is used to clean the surface in engineering purposes. It provides improvement in thermal performance, which play important role in power generation, hyperthermia, micro fabrication, air conditioning and metallurgical field. Magnesium oxide *MgO* compound consists of *Mg*^2+^ and *O*^2−^ ions, together bonded by strong ionic bond which can be synthesized by the calcination of magnesium hydroxide *Mg (OH)*^2^ and *MgCO*^3^ (magnesium carbonate) at 700–1500 °C. It is mostly efficacious for refractory and electrical applications. Similarly, the antibacterial upshots of silver *Ag* nano-size particles have been used to manage the bacterial growth in a several applications, such as dental work, burns and wound treatment, surgery applications and biomedical apparatus. The silver-based compounds and silver ions are highly toxic to microorganisms. Hussanan et al.^14^ examined the Oxide nanoparticles for the up gradation of energy in engine nanofluids, kerosene oil and water. Acharya and Mabood^15^ have studied the hydrothermal characteristics of both common nanoliquid and hybrid nanoliquid flow over a permeable slippery bent surface using Runge Kutta fourth order Method RK-4. The heat transmission and flow pattern in presence of solar radiation of hybrid nanoliquid for several solar thermal apparatus is revealed by^16^. They considered Copper-Alumina nano-ingredients with the base fluid. To refine the heat transmission in an inclined cavity Motlagh and Soltanpour^17^ used $Al_{2} O_{3}$ Aluminum Oxide. The size, type, preparation method, dispersibility of nanoparticles, compatibility and purity of base fluid and nanoparticle greatly affects the thermal properties of nanofluids. The most common used nanoparticles in base fluids are metal oxides $Fe_{2} O_{3}$, *MgO*, $Al_{2} O_{3}$, $TiO_{3}$, *CuO*, metal nitride *AIN*, Carbon nanotubes and metal like (*Au, Ag, Ni, Cu*) etc. Acharya et al.^18^ scrutinized the hybrid nanoliquid flow with the Hall current characteristics under the magnetic and thermal radiation effects over a spinning disk. They considered an innovative class of nanoliquid consists of Titanium Dioxide (*TiO*2) and Copper (Cu) nanoparticles. The magnetic effect on the flow with SWCNTs and MWCNTs over a moving/static wedge in a permeable channel was calculated by Akber et al.^19^. Some relevant literature related to the present work is present in^20,21^.

The perturbation methods are mostly used for the solution of non-linear problems, to find its approximate solutions. However, it depends on small/large parameters, due to which it cannot be widely used. For the non-linear problems an analytic tool (HAM) was introduced by Liao^22^, which is based on topology concept^23^. The HAM (Homotopy Analysis Method) has many advantages such as, it provides us more convenient way than any other analytic method to control the series solution convergence and even it can be applied to those problems, which do not have any small/large parameters^24^. This technique has been already used for several non-linear problems to obtain its analytic solutions^25^. Muhammad et al.^26^ studied the entropy generation, thermal and momentum proclamation on boundary layer flow over a linear surface using HAM technique. The MHD (magnetohydrodynamic) flow of nanoliquid over a spinning disk consist of silver *Ag* particles, with variable thickness using HAM procedure is scrutinized by Doh et al.^27^. They presumed that the all the velocities of rotating surface rises with positive increment in disk thickness. Asifa et al.^10^ highlighted the fine point of CNTs hybrid nanoliquid flow over revolving surface using HAM technique. They noticed that, the growing credit of disk rotation significantly accelerate the heat transmission rate and fluid velocity. The steady magnetic flow of nanoliquid via a porous surface with slip conditions and viscous dissipation by employing HAM technique is discussed by Alreshidi et al.^28^.

**Figure 1:**
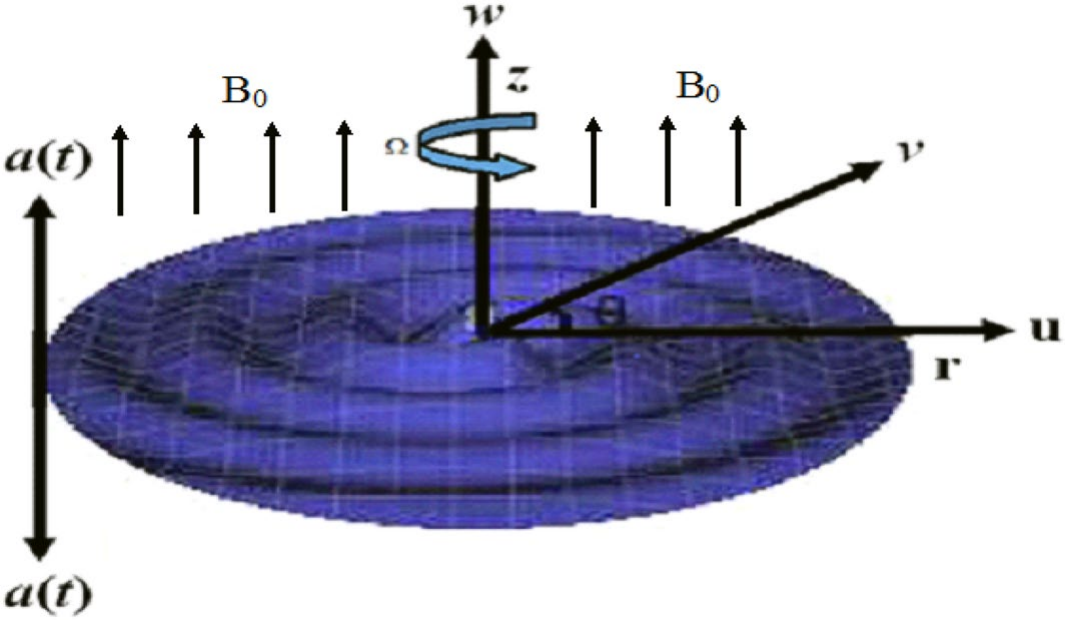
Wavy disk.

The ambition of consideration is to extend the idea of Ref.^29^ and to investigate the effect of two different nanoparticle Silver Ag and magnesium oxide MgO/Water hybrid nanoliquids over a wavy rotating disk, with upward/downward movement. To improve the thermal conductivity of the fluid flow, this study is taken under consideration. The modeled equations are solved analytically via HAM and for validation and comparison purpose of the outcomes, the Parametric continuation method (PCM) has been implemented. Both results manifest best consensus with each other (Fig. 1).

## Mathematical formulation

This section will explain the physical interpretation of the problem, thermophysical properties and equation of motion.

### Physical description of the problem

Let us consider a three-dimensional flow of Silver magnesium oxide hybrid Ag-MgO/Water nanoliquid over upward/downward moving wavy rotating disk. At time $t$, the disk has a vertical velocity $\omega = a\left( t \right)$ and is at a vertical distance $Z = a\left( t \right)$. The disk was $a\left( 0 \right) = h$ at $t = 0$. The rotating disk has angular velocity $\Omega \left( t \right)$ about $z{\text{ } - \text{ axis}}$, the buoyancy effects are negligible and it has been assumed that the nanoparticle are distributed consistent and be in equilibrium state. The uniform magnetic field of constant magnitude $\vec{B} = \left( {B_{r} \vec{e}_{r} + B_{\theta } \vec{e}_{\theta } } \right)$ and $B = \sqrt {B_{r}^{2} + B_{\theta }^{2} }$ is applied respectively, where $\vec{e}_{r}$ and $\vec{e}_{\theta }$ are unit vectors.

### Thermophysical properties of nanoliquid

The specific heat capacity and the density of the hybrid nanoliquid can be expounded are as follow^29^:

1 $$ \left( {\rho C_{p} } \right)_{hnf} = \left\{ {\left( {1 - \varphi_{2} } \right)\left[ {\left( {1 - \varphi_{1} } \right)\left( {\rho C_{p} } \right)_{f} + \varphi_{1} \left( {\rho C_{p} } \right)_{s1} } \right]} \right\} + \varphi_{2} \left( {\rho C_{p} } \right)_{s2} , $$

2 $$ \rho_{hnf} = \left\{ {\left( {1 - \varphi_{2} } \right)\left[ {\left( {1 - \varphi_{1} } \right)_{{\rho_{f} }} + \varphi_{1} \rho_{s1} } \right]} \right\} + \varphi_{2} \rho_{s2} , $$

where $\rho_{s1} ,\rho_{s2}$ are the density, $\left( {C_{p} } \right)_{s1} ,\left( {C_{p} } \right)_{s2}$ are specific heat capacity and $\varphi_{1} ,\varphi_{2}$ are the volume fraction of the silver and magnesium oxide nanoliquid respectively, which are mentioned in Table 1.

**Table 1:** Numerical properties of the water and hybrid nanofluid ^29^.

	$\rho (kg/m^{3} )$	$C_{p} (j/kgK)$	$k(W/mK)$	$\beta \times 10^{5} \left( {K^{ - 1} } \right)$
Pure water	997.1	4179	0.613	21
Magnesium oxide	3560	955	45	1.80
Silver	10,500	235	429	1.89

The viscosity $\mu_{hnf}$ of nanofluid is calculated by curve fitting on real experimental data^29^.

3 $$ \mu_{hnf} = \left( {1 + 2.5\varphi_{1} + 6.2\varphi_{2}^{2} } \right)\mu_{f} . $$

Here, the Prandtl number and the thermal conductivity of nanoliquid are defined as^29^:

4 $$ \Pr_{hnf} = \frac{{\left( {\mu C_{p} } \right)_{hnf} }}{{k_{hnf} }}, \, k_{hnf} = - \frac{{q_{w} }}{\partial \theta /\partial y}. $$

### Equation of motion

The governing equation for unsteady, incompressible, MHD forced convective flow is defined as^29,30^:

5 $$ \frac{\partial u}{{\partial r}} + \frac{\partial w}{{\partial z}} + \frac{u}{r} = 0, $$

6 $$ \rho_{hnf} \left( {\frac{\partial u}{{\partial t}} + u\frac{\partial u}{{\partial r}} + w\frac{\partial u}{{\partial z}} - \frac{{v^{2} }}{r}} \right) = - \frac{\partial p}{{\partial r}} + \mu_{hnf} \left( {\frac{{\partial^{2} u}}{{\partial r^{2} }} + \frac{{\partial^{2} u}}{{\partial z^{2} }} + \frac{1}{r}\frac{\partial u}{{\partial r}} - \frac{u}{{r^{2} }}} \right) + F_{r} , $$

7 $$ \rho_{hnf} \left( {\frac{\partial v}{{\partial t}} + u\frac{\partial v}{{\partial r}} + w\frac{\partial v}{{\partial z}} - \frac{uv}{r}} \right) = \mu_{hnf} \left( {\frac{{\partial^{2} v}}{{\partial r^{2} }} + \frac{{\partial^{2} v}}{{\partial z^{2} }} + \frac{1}{r}\frac{\partial v}{{\partial r}} - \frac{v}{{r^{2} }}} \right), $$

8 $$ \rho_{hnf} \left( {\frac{\partial w}{{\partial t}} + u\frac{\partial w}{{\partial r}} + w\frac{\partial w}{{\partial z}}} \right) = - \frac{\partial p}{{\partial r}} + \mu_{hnf} \left( {\frac{{\partial^{2} w}}{{\partial r^{2} }} + \frac{{\partial^{2} w}}{{\partial z^{2} }} + \frac{1}{r}\frac{\partial w}{{\partial r}}} \right) + F_{\theta } , $$

9 $$ \left( {\frac{\partial T}{{\partial t}} + u\frac{\partial T}{{\partial r}} + w\frac{\partial T}{{\partial z}}} \right) = \frac{k}{{\left( {\rho C_{p} } \right)_{hnf} }}\left( {\frac{{\partial^{2} T}}{{\partial r^{2} }} + \frac{1}{r}\frac{\partial T}{{\partial r}} + \frac{{\partial^{2} T}}{{\partial z^{2} }}} \right). $$

Here body forces $F_{r}$ along *x* and $F_{\theta }$ along $z$ direction respectively. It can be expressed as^29^:

10 $$ F_{r} = \frac{{Ha^{2} \mu_{hnf} }}{{R^{2} }}\left( {v\sin \theta \cos \theta - u\sin^{2} \theta } \right), $$

11 $$ F_{\theta } = \frac{{Ha^{2} \mu_{hnf} }}{{R^{2} }}\left( {u\sin \theta \cos \theta - v\sin^{2} \theta } \right). $$

Here $u,\,v,\,w$ is the velocity component of the fluid, while Ha is $LB_{0} \sqrt {\frac{\sigma }{\mu }} ,\,$ in which $B_{0}$ is the magnitude and $\theta$ is the direction of magnetic field.

### Boundary condition

The initial and boundary condition for wavy spinning disk are:

$$ u = 0, \, v = r\Omega_{0} \left( t \right), \, w = w_{0} \left( t \right), \, T = T_{0} ,{\text{ at }}z = 0 $$

12 $$ u \to 0, \, v \to 0, \, w \to 0, \, T \to T_{\infty } ,{\text{ at }}z \to \infty . $$

### Karman’s approach

In order to transform the Eqs. (5–9) and (12) to the system of ODEs, we use the following transformation, we follow^30^.

$$ u = \frac{rv}{{a^{2} \left( t \right)}}f\left( \eta \right), \, \,v = \frac{rv}{{a^{2} \left( t \right)}}g\left( \eta \right),\, \, w = \frac{v}{a\left( t \right)}h\left( \eta \right), \, p = \frac{{pv^{2} }}{{a^{2} \left( t \right)}}p\left( \eta \right), $$

13 $$ T = T_{\infty } + \Delta T_{\infty } , \, \,\eta = \frac{Z}{a\left( t \right)} - 1,\, \, \eta_{Z} = \frac{1}{a\left( t \right)}, \, \, \, \eta_{t} = \frac{ - a\left( t \right)}{{a\left( t \right)}}\left( {\eta + 1} \right). $$

The following system of ordinary differential equation is formed by using Eq. (13) in Eqs. (5–9):

14 $$ f^{\prime\prime} = \frac{{\rho_{hnf} }}{{\mu_{hnf} }}\left( {hf^{\prime} + f^{2} - g^{2} - S\frac{{\left( {\eta + 1} \right)f^{\prime}}}{2} + f} \right) + A\omega \left( {g\sin \theta \cos \theta - f\sin^{2} \theta } \right), $$

15 $$ g^{\prime\prime} = \frac{{\rho_{hnf} }}{{\mu_{hnf} }}\left( {hg^{\prime} + 2fg - S\left( {\frac{{\left( {\eta + 1} \right)g^{\prime}}}{2} - g} \right)} \right), $$

16 $$ h^{\prime\prime} = \frac{{\rho_{hnf} }}{{\mu_{hnf} }}\left( {hh^{\prime} - S\frac{{\left( {\eta + 1} \right)h^{\prime}}}{2} + h^{\prime}} \right) - A\omega \left( {f\sin \theta \cos \theta - g\sin^{2} \theta } \right), $$

17 $$ \theta^{\prime\prime} = \rho_{hnf} \left( {h\theta^{\prime} - S\left( {\frac{{\left( {\eta + 1} \right)\theta^{\prime}}}{2} + \gamma \theta } \right)} \right). $$

The diminished conditions are:

$$ f\left( 0 \right) = 0, \, h\left( 0 \right) = \beta \frac{S}{2}, \, g\left( 0 \right) = \omega , \, \theta \left( 0 \right) = 1,{\text{ at }}\eta = 0, $$

18 $$ f\left( \eta \right) \to 0, \, g\left( \eta \right) \to 0, \, h\left( \eta \right) \to 0, \, \theta \left( \eta \right) \to 0,{\text{ as }}\eta = \infty . $$

Since the physical constraint *S* controlling the up/down movement of the disk (or the contraction/expansion of the disk) is defined as^30^:

19 $$ S = 2\frac{{a^{*} \left( t \right)a\left( t \right)}}{v}, $$

Sign $\omega$ nominate the constant rotation of wavy disk^30^:

20 $$ \omega = 2\frac{{a^{2} \left( t \right)\Omega \left( t \right)}}{v}. $$

And disk temperature parameter, which express temperature distribution:

21 $$ \gamma = \frac{1}{2}\frac{a\left( t \right)T}{{a^{*} \left( t \right)\Delta T}}. $$

The non-dimensional form of Nusselt number and skin friction are expressed as:

22 $$ Nu = \frac{{rq_{w} }}{{k_{f} \left( {T_{w} - T_{\infty } } \right)}}\,\,\,\,{\text{and}}\,\,\,C_{f} = \frac{{\sqrt {\tau_{wr}^{2} - \tau_{w\phi }^{2} } }}{{\rho_{f} \left( {\Omega \,r} \right)^{2} }}. $$

where, $\tau_{w\phi }$ and $\tau_{w\,r}$ stand for transverse and radial stress respectively.

## Problem solution

The analytical approach HAM, which was presented by Liao^22–24^ has been used for the solution of nonlinear modeled differential equations. For strong convergence, BVP 2.0 package has been implementing to show sum of square residual error.

The linear operators $\pi_{f}$,$\pi_{g}$,$\pi_{h}$ and $\pi_{\Theta }$ are presented as,

17 $$ \pi_{f} = \frac{{\partial^{2} f}}{{\partial \eta^{2} }},\,\,\,\,\pi_{g} = \frac{{\partial^{2} g}}{{\partial \eta^{2} }},\,\,\,\,\pi_{h} = \frac{{\partial^{2} h}}{{\partial \eta^{2} }},\,\,\,\,\,\pi_{\theta } = \frac{{\partial^{2} \theta }}{{\partial \eta^{2} }}. $$

The expand form of $\pi_{f}$,$\pi_{g}$,$\pi_{h}$ and $\pi_{\Theta }$ are ,

22 $$ \pi_{f} (\chi_{1} + \chi_{2} \eta ) = 0,\,\,\,\pi_{g} (\chi_{3} + \chi_{4} \eta ) = 0,\,\,\,\pi_{h} (\chi_{5} + \chi_{5} \eta ) = 0\,\,{\text{and}}\,\,\,\pi_{\Theta } (\chi_{4} + \chi_{5} \xi ) = 0. $$

Taylor’s series expansion form is used

23 $$ f(\eta ;\rho ) = f_{0} (\eta ) + \sum\limits_{x = 1}^{\infty } {f_{x} (\eta )\rho^{x} ,} $$

24 $$ g(\eta ;\rho ) = g_{0} (\eta ) + \sum\limits_{x = 1}^{\infty } {g_{x} (\eta )\rho^{x} ,} $$

25 $$ h(\eta ;\rho ) = h_{0} (\eta ) + \sum\limits_{x = 1}^{\infty } {h_{x} (\eta )\rho^{x} ,} $$

26 $$ \theta (\xi ;\rho ) = f_{0} (\xi ) + \sum\limits_{x = 1}^{\infty } {\theta_{x} (\xi )\rho^{x} ,} $$

Now

27 $$ \begin{gathered} f_{x} (\eta ) = \frac{1}{x}\frac{df(\eta ;\rho )}{{d\eta }}|_{\rho = 0} ,\,\,\,\,\,g_{x} (\eta ) = \frac{1}{x}\frac{dg(\eta ;\rho )}{{d\eta }}|_{\rho = 0} {,}\,\,\,h_{x} (\eta ) = \frac{1}{x}\frac{dh(\eta ;\rho )}{{d\eta }}|_{\rho = 0} \,, \hfill \\ \,\theta_{x} (\eta ) = \frac{1}{x}\frac{d\theta (\eta ;\rho )}{{d\eta }}|_{\rho = 0} . \hfill \\ \end{gathered} $$

The system of equation can be written in the form of:

28 $$ L_{f} \left[ {f_{x} (\eta ) - N_{x} f_{x - 1} (\eta )} \right] = \pi_{f} R_{x}^{f} (\eta ), $$

29 $$ L_{g} \left[ {g_{x} (\eta ) - N_{x} g_{x - 1} (\eta )} \right] = \pi_{g} R_{x}^{g} (\eta ), $$

30 $$ L_{h} \left[ {h_{x} (\eta ) - N_{x} h_{x - 1} (\eta )} \right] = \pi_{h} R_{x}^{h} (\eta ), $$

31 $$ L_{\theta } \left[ {\theta_{x} (\eta ) - N_{x} \theta_{x - 1} (\eta )} \right] = \pi_{\theta } R_{x}^{\theta } (\eta ), $$

where $N_{x} = 0$ if $\rho \le 1$ and if $\rho > 1$.

## Result and discussion

**Figure 2:**
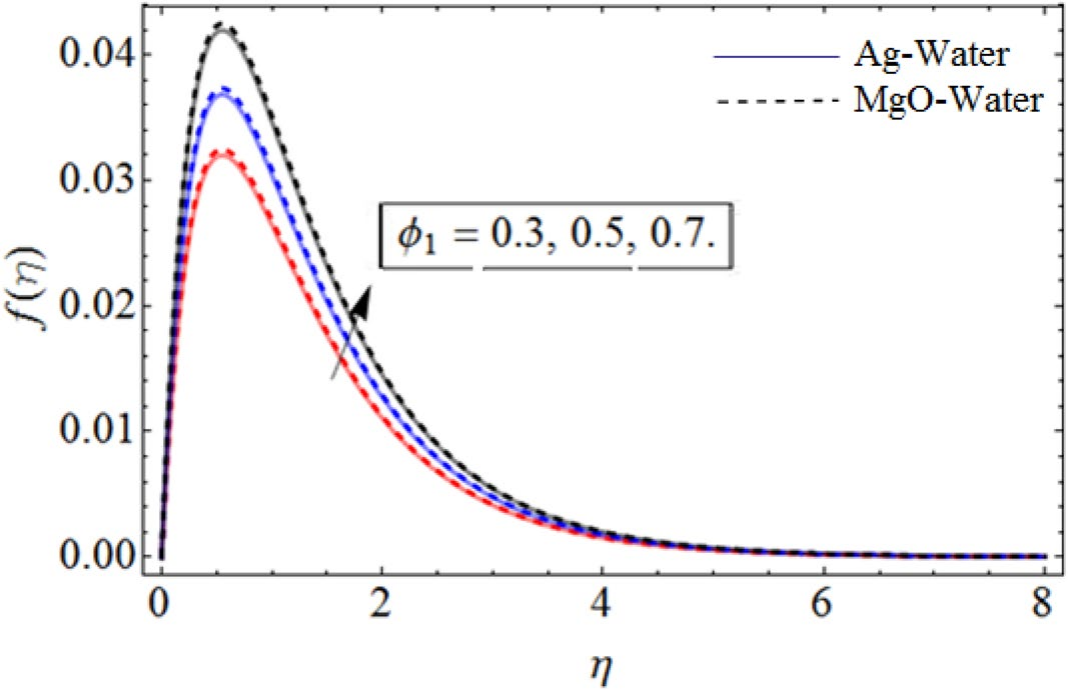
$\phi_{1}$ out-turn versus axial velocity $f\left( \eta \right)$. When $\Pr = 6.7,\beta = 0.7,\phi_{2} = 0.9,S = 2.2,\omega = 1.0.$

**Figure 3:**
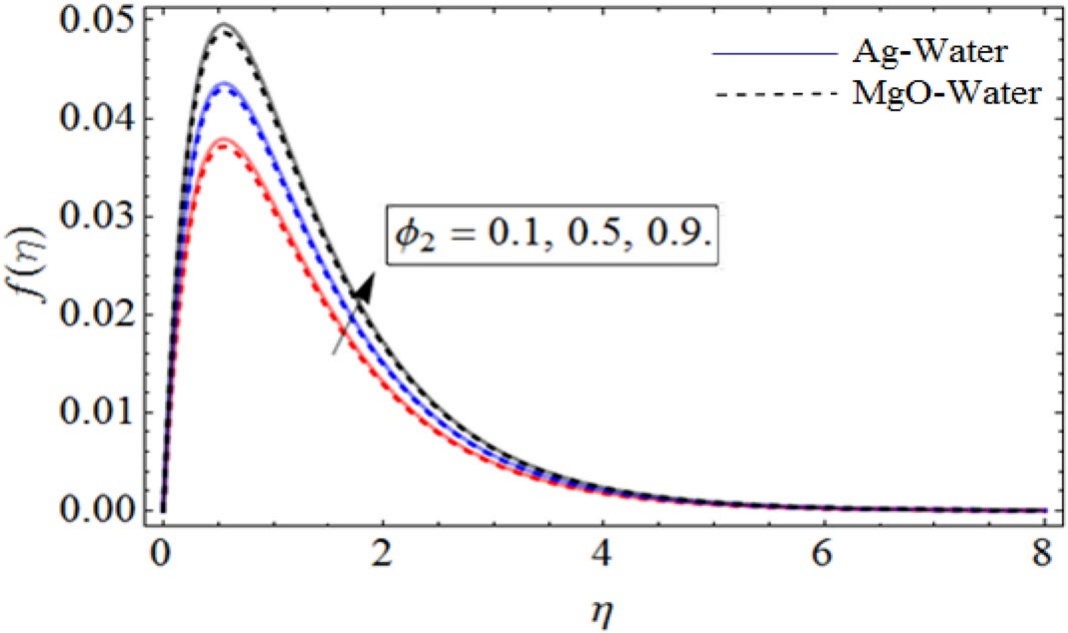
$\phi_{2}$ out-turn versus the axial velocity $f\left( \eta \right)$. When $\Pr = 6.7,\,\,\beta = 0.7,\phi_{1} = 0.7,S = 2.2,\omega = 1.0.$

**Figure 4:**
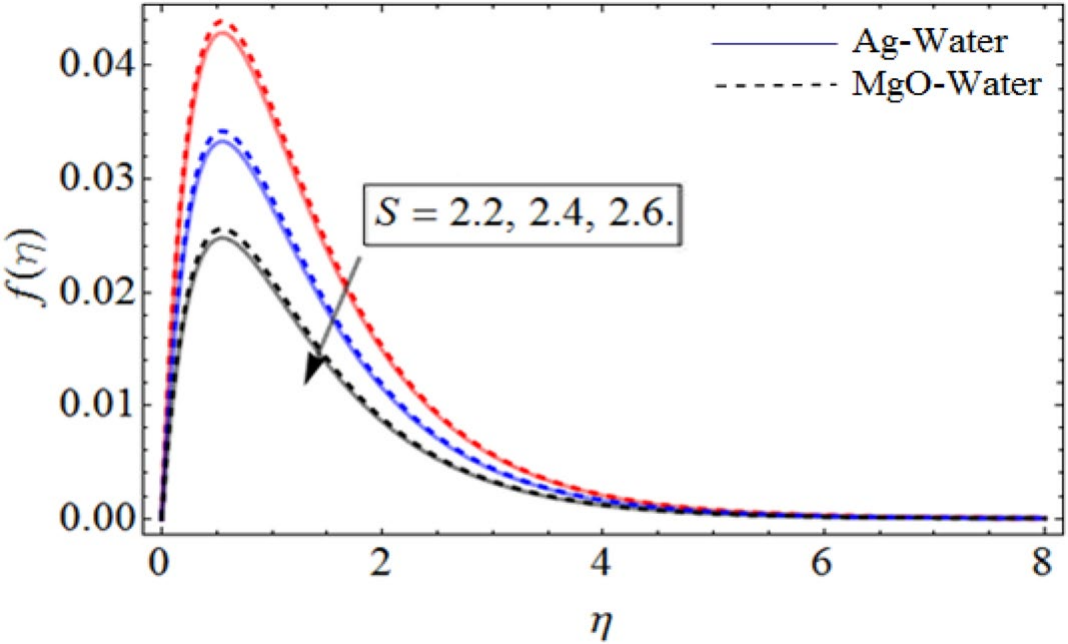
*S* out-turns versus the axial velocity $f\left( \eta \right)$. When $\Pr = 6.7,\beta = 0.7,\phi_{1} = 0.7,\phi_{2} = 0.9,\omega = 1.0.$

**Figure 5:**
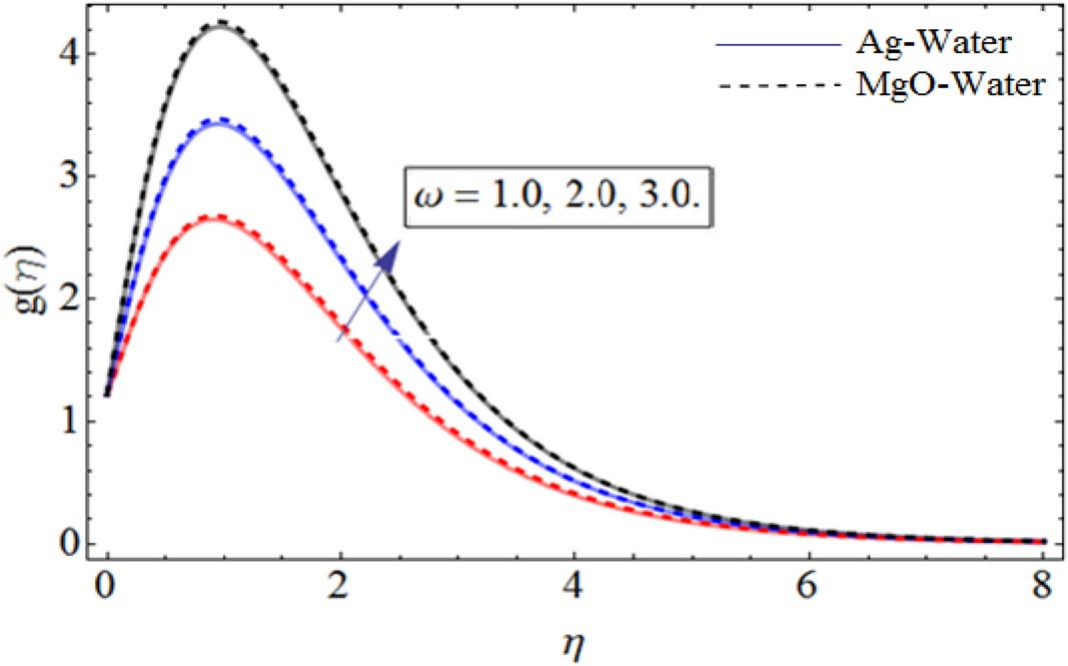
$\omega$ out-turn versus the radial velocity $g\left( \eta \right)$ When $\Pr = 6.7,\gamma = 0.3,\beta = 0.7,\phi_{1} = 0.7,\phi_{2} = 0.9.$

**Figure 6:**
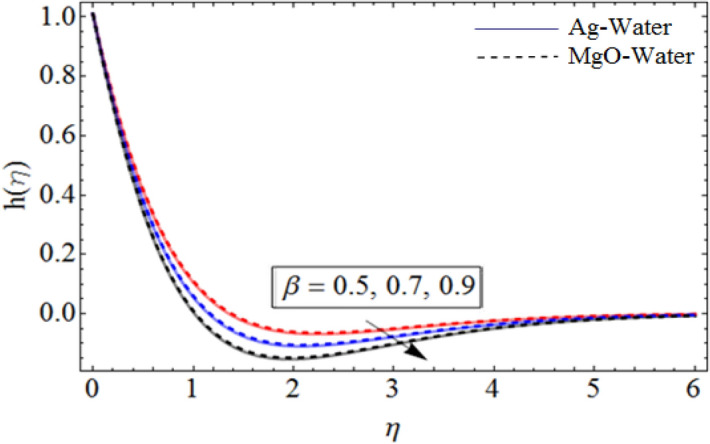
$\beta$ out-turn versus the azimuthal velocity $h\left( \eta \right)$. When $\Pr = 6.7,\phi_{1} = 0.7,\phi_{2} = 0.9,S = 2.2,\omega = 1.0.$

**Figure 7:**
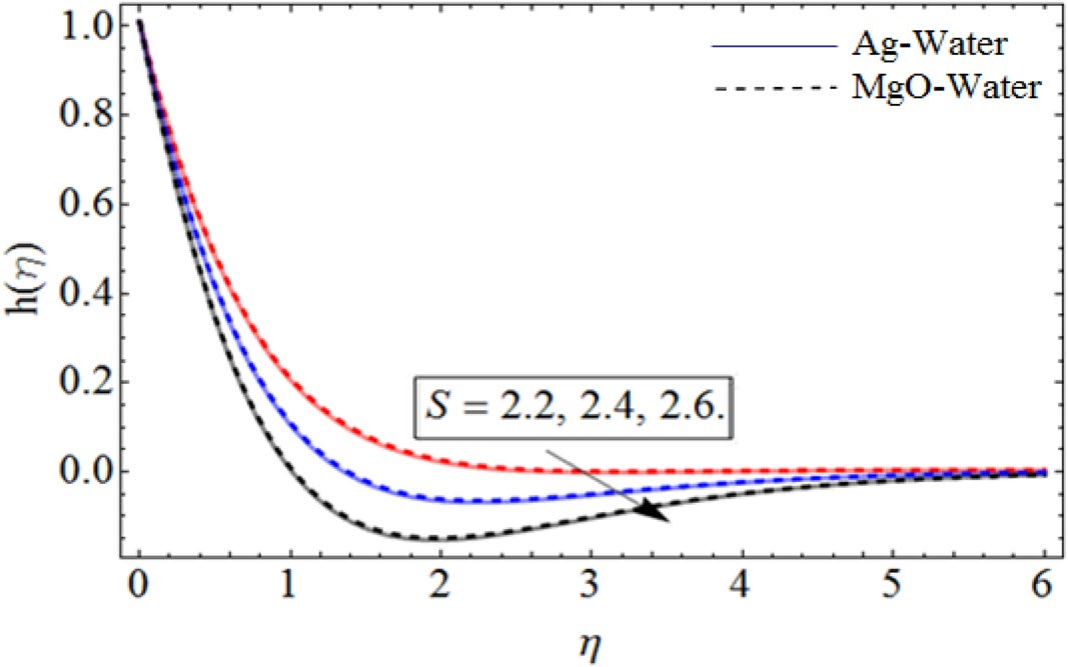
*S* out-turns versus the azimuthal velocity $h\left( \eta \right)$. When $\Pr = 6.7,\beta = 0.7,\phi_{1} = 0.7,\phi_{2} = 0.9,\omega = 1.0.$

**Figure 8:**
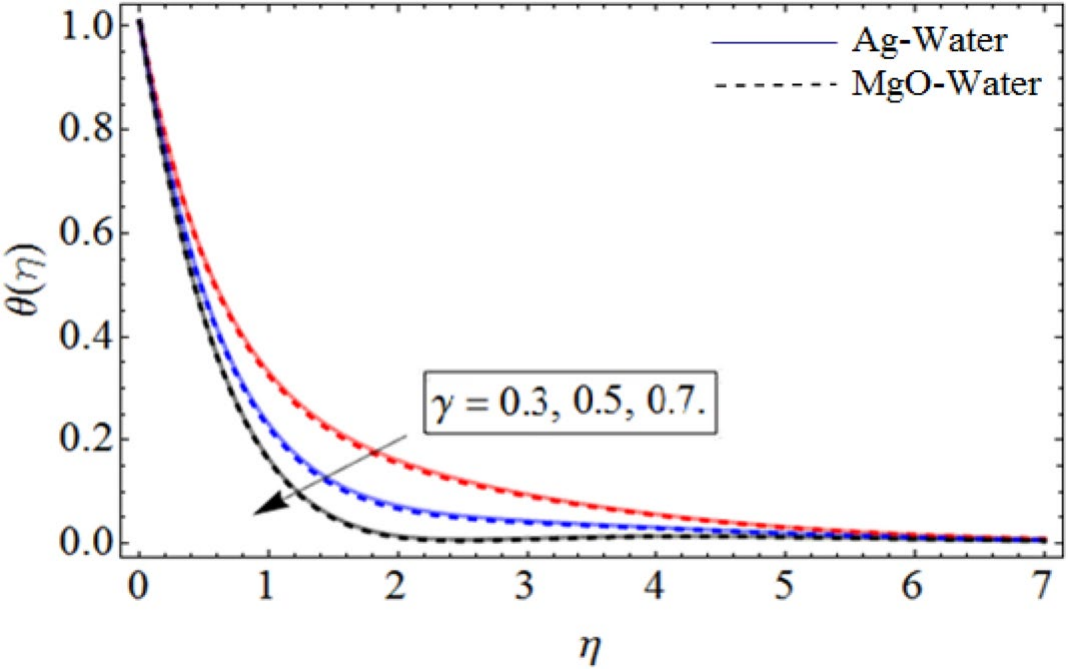
$\gamma$ out-turn versus temperature profile $\theta \left( \eta \right)$. When $\Pr = 6.7,\beta = 0.7,\phi_{1} = 0.7,\phi_{2} = 0.9,\omega = 1.0.$

**Figure 9:**
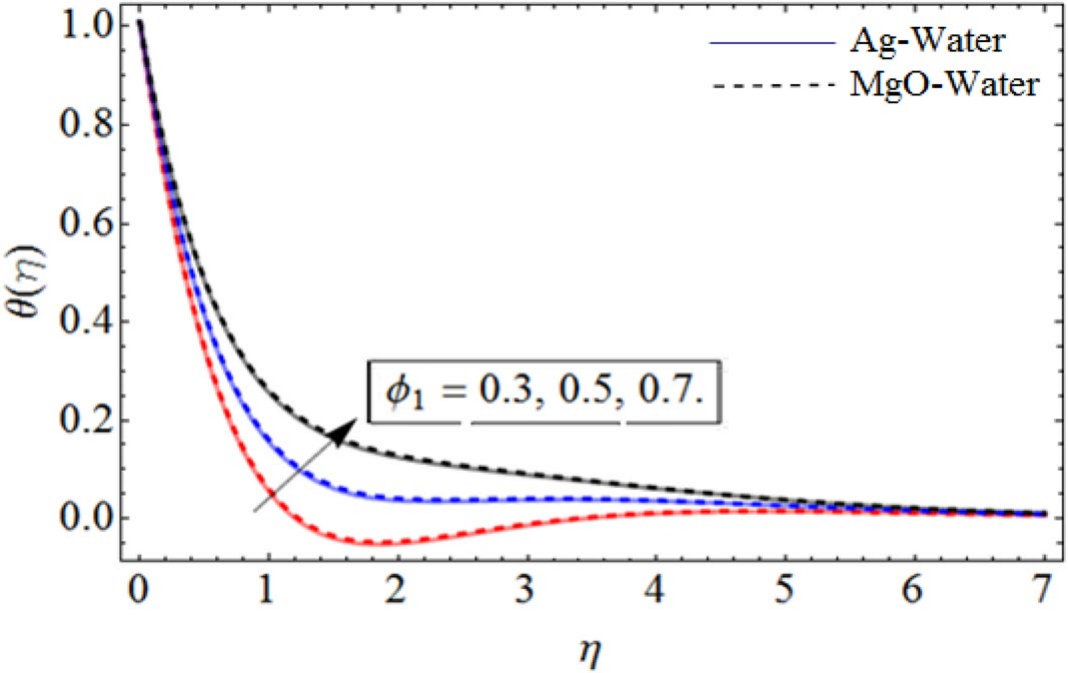
$\phi_{1}$ out-turn versus the temperature profile $\theta \left( \eta \right)$. When $\Pr = 6.7,\beta = 0.7,\phi_{2} = 0.9,S = 2.2,\omega = 1.0.$

**Figure 10:**
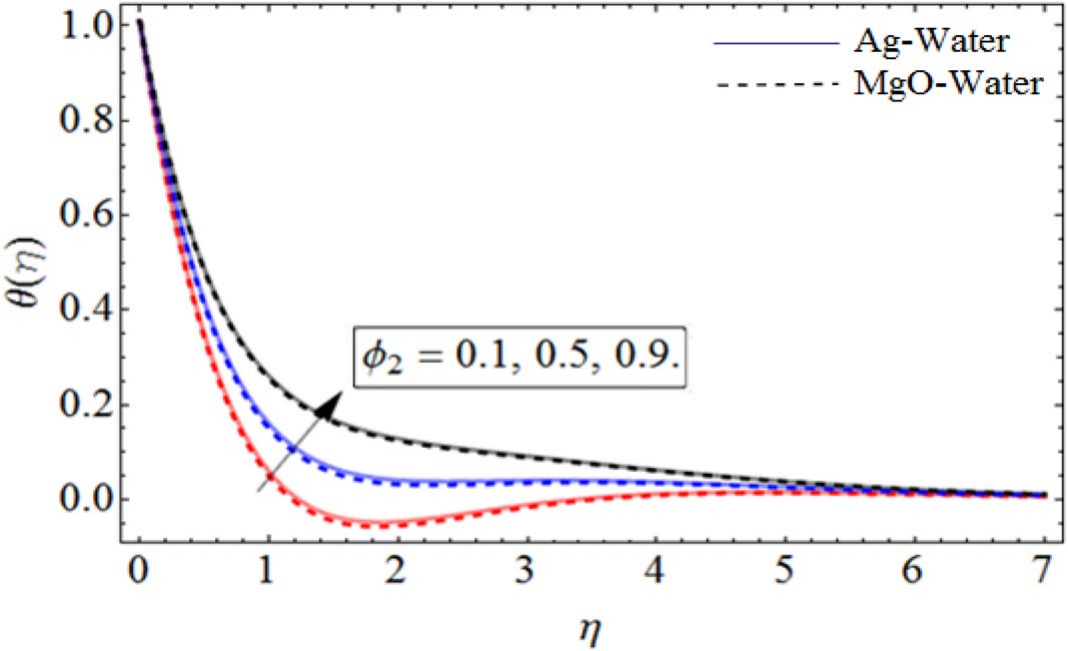
$\phi_{2}$ out-turn versus the temperature $\theta \left( \eta \right)$. When $\Pr = 6.7,\beta = 0.7,\phi_{1} = 0.7,S = 2.2,\omega = 1.0.$

**Figure 11:**
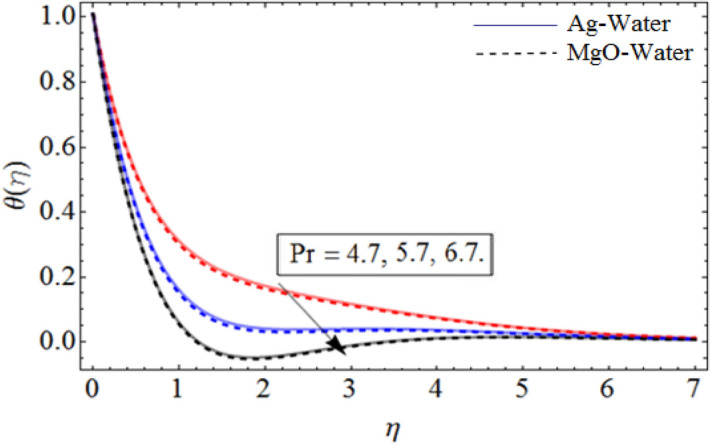
*Pr* out-turn versus the temperature $\theta \left( \eta \right)$. When $\beta = 0.7,\phi_{1} = 0.7,\phi_{2} = 0.9,S = 2.2,\omega = 1.0.$

The time dependent, 3D hybrid nanoliquid flow over a wavy rotating disk with upward/downward motion has been studied. The numerical results of the system of differential equations has been acquire through Parametric continuation method (PCM), while for comparison and validity of results and to get analytical output, HAM technique has been applied. The effect of physical parameter has been shown in Figs. 2, 3, 4, 5, 6, 7, 8, 9, 10 and 11. For comparative studies of PCM and HAM, Tables 2, 3 and Tables 4 are plotted.

**Table 2:** Numerical comparison of PCM method versus HAM for the physical parameters $S = 2.1,$ $\Pr = 2.7,$ $\gamma = 0.04,$ $\phi_{1} = 1.1,$ $\phi_{2} = 0.75,$ $A = 1.2,$ $W = 0.04$ and $B = 0.3.$

$\eta$	PCM (numerical results)			HAM (analytical results)		
	$f\left( \eta \right)$	$g\left( \eta \right)$	$\theta \left( \eta \right)$	$f\left( \eta \right)$	$g\left( \eta \right)$	$\theta \left( \eta \right)$
0.0	0.0000	0.0000	1.0000	0.0000	0.0000	1.0000
0.5	0.0001	0.0010	0.2811	0.0001	0.0010	0.2821
1.0	0.0050	0.0147	0.0562	0.00051	0.01478	0.0562
1.5	− 0.0381	− 0.0761	0.0083	− 0.0383	− 0.0765	0.0087
2.0	− 0.1369	− 0.1903	0.0009	− 0.1370	− 0.1904	0.0009

**Table 3:** It shows the comparative behavior of *Ag* and *MgO* on radial and tangential velocities $\left( {f^{\prime}\left( 0 \right),\,g^{\prime}\left( 0 \right)} \right)$ for volume fraction parameter.

$\eta$	Silver (Ag)		Magnesium oxide $MgO$	
	$f^{\prime}\left( 0 \right)$	$g^{\prime}\left( 0 \right)$	$f^{\prime}\left( 0 \right)$	$g^{\prime}\left( 0 \right)$
0.00	1.4725	1.6912	1.6323	1.7562
0.05	1.6134	1.7724	1.9735	1.8821
0.01	1.8642	1.9054	2.2012	1.9922
0.15	2.0500	2.1791	2.3700	1.3901
0.20	2.3531	2.4753	2.5531	2.6102

**Table 4:** The numerical output of skin fraction and Nusselt number $h^{\prime}\left( 0 \right),\theta^{\prime}\left( 0 \right)$.

$\eta$	Silver (Ag)		Magnesium oxide $MgO$	
	$h^{\prime}\left( 0 \right)$	$\theta^{\prime}\left( 0 \right)$	$h^{\prime}\left( 0 \right)$	$\theta^{\prime}\left( 0 \right)$
0.00	1.3724	1.7954	1.6513	1.7582
0.05	1.3921	1.5362	1.4835	1.8734
0.10	1.6139	1.3482	0.2012	1.8622
0.15	0.4612	1.8014	1.4910	2.4684
0.20	0.5917	1.7961	2.7893	2.6212

Figure 1 displays the hybrid nanofluid flow over a wavy spinning disk under the magnetic effects. Figures 2 and 3 demonstrate the influence of volume fraction parameter $\phi_{1}$ or $\phi_{Ag}$ and $\phi_{2}$ or $\phi_{MgO}$ on axial velocity profile $f\left( \eta \right)$. It shows that, by increasing the number of silvers $Ag$ and magnesium oxide nanoparticles, the axial velocity of fluid significantly improve. Figure 4 highlights the dominance of unsteadiness parameter *S* versus axial velocity $f\left( \eta \right)$**.** The rising credit of *S* declines the fluid velocity. Figure 5 depicts the out-turn of rotation parameter $\omega$ on the radial velocity $g\left( \eta \right)$. It can be presumed that, the increment in $\omega$ increases the kinematic energy of the fluid particles, consequently, the velocity accumulates, which generate some amount of heat. Eventually, the disk surface becomes heated, which also improve the fluid temperature $\theta \left( \eta \right)$.

Figures 6 and 7 show the upshot of $\beta$ and controlling parameter $S$ on the azimuthal velocity respectively. As $S$ control the movement of the spinning disk, when we increases the values of $S$, the rate of upward/downward motion of the disk also increases. So, the inter-molecular forces between the fluid particles become week, and during the upward motion of the disk the fluid molecule loses its energy, which causes the decline of temperature and azimuthal velocity as well.

**Figure 12:**
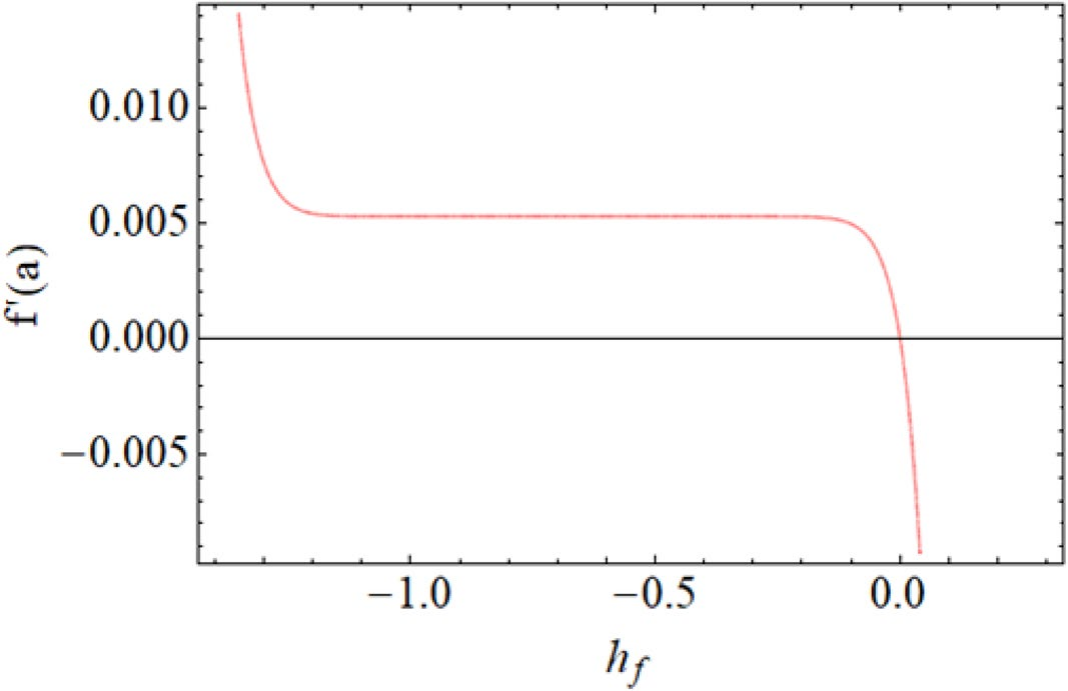
$$h_{f}$$ When $$\beta = 0.7,\phi_{1} = 0.7,\phi_{2} = 0.9,S = 2.2,\omega = 1.0.$$

**Figure 13:**
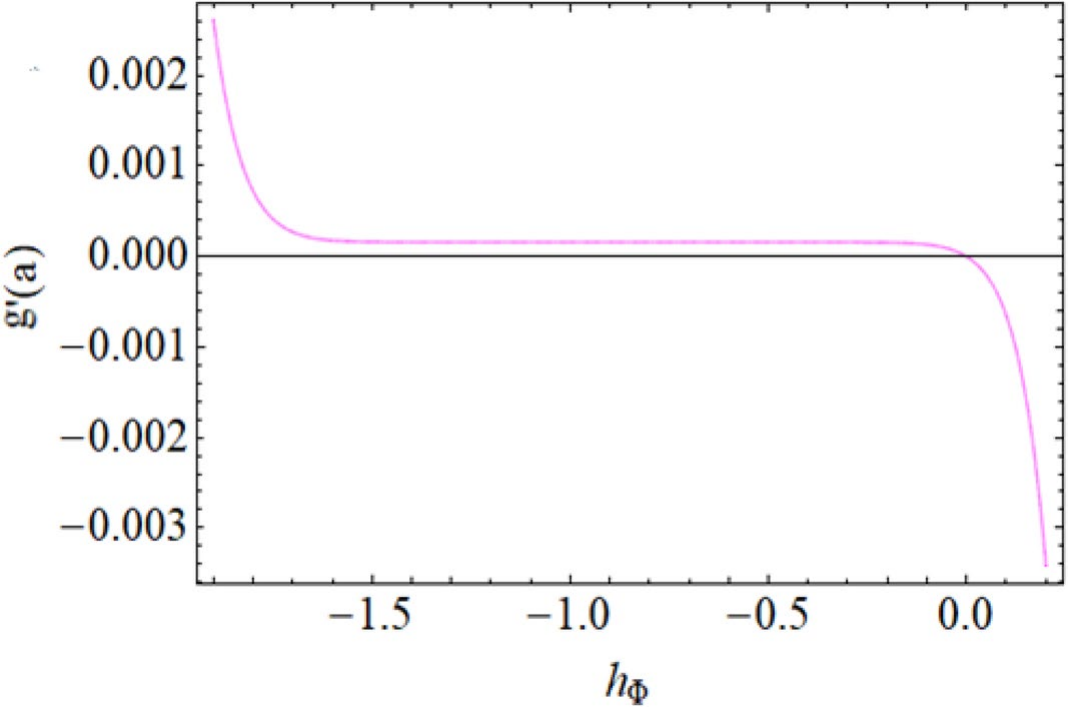
$$h_{g}$$ When $$\beta = 0.7,\phi_{1} = 0.7,\phi_{2} = 0.9,S = 2.2,\omega = 1.0.$$

**Figure 14:**
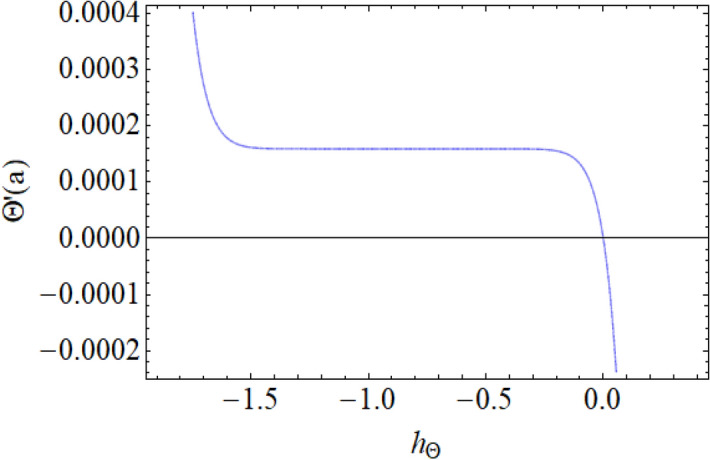
$$h_{\Theta }$$ When $$\beta = 0.7,\phi_{1} = 0.7,\phi_{2} = 0.9,S = 2.2,\omega = 1.0.$$

Figure 8 depict the effects of $\gamma$ on temperature. Parameter $\gamma$ actually controls the upward and downward velocity of spinning disk. So, from Fig. 8, we can presume that the increasing values of $\gamma$ will reduced the hybrid nanofluid temperature. Figures 9 and 10 illustrate the influence of $\phi_{1}$ and $\phi_{2}$ on temperature profile $\theta \left( \eta \right)$ respectively. As the volume fraction of silver and magnesium $MgO$ nanoparticles increase, the heat absorbing ability of fluid also increased, which result in enhancement of fluid temperature $\theta \left( \eta \right)$. Figure 9 shows the decreases of temperature versus increases in Prandtl number. $\Pr_{hnf} = \left( {\mu C_{p} } \right)_{hnf} /k_{hnf}$, physically less Prandtl fluid has higher thermal diffusivity. The thermal boundary layer thickness reduces with larger values of Prandtl number as a result in decrease of the temperature. Figures 12, 13, and 14 revealed the $h$-curves for axial velocity $h_{f}$, radial velocity $h_{g}$ and temperature $h_{\Theta }$ fields respectively.

Table 2 illustrates the numerical comparison of PCM method against HAM approach for radial, azimuthal, and axial velocity. From Table 2, it can be observed that fractional model show fast converges than Runge Kutta order 4 method.

Table 3 shows comparative effect of volume fraction parameter $\phi$ on radial and tangential velocities $\left( {f^{\prime}\left( 0 \right),g^{\prime}\left( 0 \right)} \right)$ for different nanofluid, keeping the rest physical parameters are constant. From, Table 3 we can examine that, the radial and tangential velocity of $MgO$ nanofluid are greater than the Silver Ag nanofluid, because the density of Silver nanoparticles are heavy than $MgO$ nanoparticles. Therefore, the viscosity of Ag nanofluid is greater than $MgO$ nanofluid. That's the reason that the radial and tangential velocity of $MgO$ nanofluid is greater.

Table 4 shows the comparative effect of volume fraction parameter $\phi$ on skin fraction and Nusselt number $h^{\prime}\left( 0 \right)$ and $\theta^{\prime}\left( 0 \right)$ for different nanofluid respectively while the rest of physical parameters are constant. The sum and square of the total residual for the *Ag* and *MgO* are displayed in Tables 5 and 6. Table 7 displays the comparison of present work with the published literature.

**Table 5:** Total squares residual errors for silver $Ag$. When $\Pr = 6.3,\,\beta = 0.3,\,\,\phi_{1} = 0.2,\,\phi_{2} = 0.4,\,\omega = 0.75.\,$

$m$	$\varepsilon_{m}^{f} \,Ag$	$\varepsilon_{m}^{g} \,Ag$	$\varepsilon_{m}^{h} \,Ag$	$\varepsilon_{m}^{\theta } \,Ag$
3	$5.15485 \times 10^{ - 3}$	$4.87652 \times 10^{ - 7}$	$5.57668 \times 10^{ - 3}$	$4.24884 \times 10^{ - 3}$
6	$3.53455 \times 10^{ - 3}$	$3.94355 \times 10^{ - 9}$	$5.1578 \times 10^{ - 3}$	$2.3572 \times 10^{ - 3}$
9	$1.48664 \times 10^{ - 4}$	$7.41788 \times 10^{ - 11}$	$4.14877 \times 10^{ - 4}$	$3.54206 \times 10^{ - 4}$
12	$6.21764 \times 10^{ - 5}$	$2.85429 \times 10^{ - 11}$	$3.1687 \times 10^{ - 5}$	$4.2456 \times 10^{ - 4}$

**Table 6:** Total squares residual errors for *MgO*. When $\Pr = 6.3,\,\beta = 0.3,\,\,\phi_{1} = 0.2,\,\phi_{2} = 0.4,\,\omega = 0.75.\,$

$m$	$\varepsilon_{m}^{f} \,MgO$	$\varepsilon_{m}^{g} \,MgO$	$\varepsilon_{m}^{h} \,MgO$	$\varepsilon_{m}^{\theta } \,MgO$
3	$1.1132 \times 10^{ - 4}$	$6.6438 \times 10^{ - 8}$	$6.6266 \times 10^{ - 4}$	$2.7428 \times 10^{ - 4}$
6	$3.5838 \times 10^{ - 4}$	$3.48569 \times 10^{ - 8}$	$4.24428 \times 10^{ - 4}$	$1.59433 \times 10^{ - 4}$
9	$2.2229 \times 10^{ - 6}$	$2.22825 \times 10^{ - 10}$	$3.14409 \times 10^{ - 5}$	$2.71522 \times 10^{ - 5}$
12	$1.69412 \times 10^{ - 5}$	$2.41559 \times 10^{ - 11}$	$1.34460 \times 10^{ - 8}$	$2.24307 \times 10^{ - 6}$

**Table 7:** The comparison of the present work with published literature.

	Duwairi^31^	Asifa et al.^10^	Present work
$f^{\prime}\left( 0 \right)$	0.625	0.62510	0.62512
− $g^{\prime}\left( 0 \right)$	− 1.708	− 1.70803	− 1.70810
− $\theta^{\prime}\left( 0 \right)$	− 2.264	− 0.78555	− 0.78557

## Conclusion

In this work, the three-dimensional, unsteady *Ag-MgO*/water hybrid nanofluid flow, caused by upward/downward movement of a wavy rotating disk, under the magnetic field influence with mass and heat transport has been studied. The following observations have been made on the basis of above computation:

- The wavy rotating surface increases the heat transmission rate up to 15%, comparatively to flat surface^32^.
- The rising credit of rotation parameter $\omega$ increases the kinematic energy of fluid, which result in the enhancement of velocity and temperature of hybrid nanoliquid.
- The fluid temperature can be control, with the addition of *Ag-MgO* nanoparticles in the base fluid.
- Magnesium oxide *MgO* compound consists of *Mg*^2+^ and *O*^2−^ ions, together bonded by strong ionic bond. Which can be synthesized at 700 °C to 1500 °C and is mostly efficacious for refractory and electrical applications
- The strong bonds between water atom (*H*^+^ + *OH*^−^) and silver ions Ag^+^ effectively improves the thermophysical properties of water.
- The upward/downward movement of wavy rotating disk positively affects the fluid temperature and velocity.
- The use of hybrid nanoliquid is more efficacious to overcome low energy transmission. Such as, it provides improvement in thermal performance of carrier fluid, which play important role in power generation, hyperthermia, microfabrication, air conditioning and metallurgical field.

## Abbreviations

*S*: Control upward/downward motion of the disk
$$T$$: Fluid temperature (K)
$\gamma$: Disk temperature parameter
$T_{\infty }$: Temperature away from the surface
*Ha*: Hartmann number
$$\left( {r,\,\theta ,\,z} \right)$$: Cylindrical coordinate
$$\eta$$: Similarity variable
$$\nu$$: Kinematic viscosity $$\left( {{\text{m}}^{2} \,{\text{s}}^{ - 1} } \right)$$
*Pr*: Prandtl number
$$\Theta$$: Dimensionless temperature
$$\sigma$$: Thermal conductivity
$\phi_{1} = \phi_{Ag}$: Volume fraction of silver
$$k_{hnf}$$: Thermal conductivity
$\rho_{s1}$: Silver specific heat capacity
$\rho_{hnf}$: Hybrid Nanofluid density
*Ag*: Silver
*PCM*: Parametric continuation method
$$u,v,w$$: Velocity component
$T_{w}$: Temperature of the Surface
$$\beta$$: Permeability parameter
$$p$$(Pa): Pressure
$\theta$: Direction of the magnetic field
*f, g, h*: Dimensional velocity
Z: Vertical distance
$$\mu$$: Dynamic viscosity $$\left( {{\text{k}}\,{\text{g}}\,\,{\text{m}}^{ - 1} {\text{s}}^{ - 1} } \right)$$
$\mu_{hnf}$: Dynamic viscosity of nanofluid
$\omega$: Velocity (vertical) of disk $\left( {{\text{r }}\,{\text{s}}^{ - 1} } \right)$
$\Omega$: Velocity (angular) of disk $\left( {{\text{r}}\,\,{\text{s}}^{ - 1} } \right)$
$\phi_{2} = \phi_{MgO}$: Magnesium oxide volume fraction
$$\alpha$$: Thermal diffusivity $$\left( {{\text{m}}^{2} \,{\text{s}}^{ - 1} } \right)$$
$\rho_{s2}$: Magnesium oxide specific heat capacity
$\nu_{hnf}$: Nanofluid kinematic viscosity
M*gO*: Magnesium oxide
HAM: Homotopy analysis method
